# Does the ST2 Level in Pediatric Heart Failure Patients Correlate with Cardiovascular Events and Mortality?

**DOI:** 10.3390/children11060718

**Published:** 2024-06-13

**Authors:** Ayse Sulu, Gulcan Uner, Pelin Kosger, Birsen Ucar

**Affiliations:** Department of Pediatric Cardiology, Faculty of Medicine, Eskisehir Osmangazi University, Eskisehir 26040, Turkey; drgulcanbilgic@gmail.com (G.U.); pkosger@ogu.edu.tr (P.K.); bucar@ogu.edu.tr (B.U.)

**Keywords:** pro BNP, heart failure, suppression of tumorigenicity 2 (ST2)

## Abstract

Introduction: The suppression of tumorigenicity 2 (ST2) is a receptor member belonging to the interleukin-1 (IL-1) family. The ligand and soluble versions are its two isoforms. The IL-33-ST2L ligand complex’s development provides protection against heart fibrosis and hypertrophy. Investigations on heart failure in adults have demonstrated that it does not change by age, body mass index (BMI), creatinine, hemoglobin, and albumin levels, in contrast to NT pro BNP. In adult heart failure patients, it has been demonstrated to be an independent predictor of mortality and cardiovascular events. The most recent guideline recommends using it as class 2b in the diagnosis of adult heart failure. Studies on ST2 in children are rare. The purpose of this study is to assess the prognostic value of ST2 for cardiovascular events in young individuals suffering from heart failure. Method: This study included pediatric patients (0–18 years old) with congenital heart disease or cardiomyopathy who needed medical care, as well as surgical or interventional treatment. Height, weight, gender, saturation, heart failure classification (Ross or NYHA), medications, the electrocardiogram, echocardiography, pro BNP, and sST2 values of the patients, as well as any hospitalization, lower respiratory tract infection, organ dysfunction, or need for angiography or surgery during follow-up data on arrhythmia and death were gathered during a 1-year follow-up. The SPSS software version 25 application was used to carry out the statistical analysis. Results: This study included 59 patients, of whom 27 (46.6%) were male. The average age of the patients was 55.5 months (1–228 months) and the average body weight was 16 kg (2.6–90 kg). Major cardiovascular events occurred in 45 of 59 patients (76.3%). Twenty-four patients experienced one MACE, while twenty-one patients experienced multiple MACEs. Pro BNP and sST2 levels were similar in the groups that developed MACE compared to those that did not. Pro BNP was discovered to be significantly higher in patients with hospitalization, growth retardation, lower respiratory tract infection, and organ failure, however, when assessing each situation (*p* = 0.001, *p* = 0.011, *p* = 0.001, *p* = 0.007, respectively). Soluble ST2 was found to be higher in patients with growth retardation than in those without (*p* = 0.037). Although the soluble ST2 level failed to demonstrate a correlation with pro BNP, it did show a positive correlation (r = 0.437) with the Ross score. When compared to other groups, it was discovered to be higher in patients with valvular insufficiency type heart disease. Conclusions: In this study, higher sST2 levels were discovered, particularly in the group with valve insufficiency and children with growth retardation. It was associated with the Ross score, but not with the pro BNP level. Although it increases in correlation with clinical heart failure, its predictive value for MACE is low. Similarly, pro BNP is not proven to be predictive; nonetheless, its high levels in patients with hospitalization, growth retardation, lower respiratory tract infection, and organ failure demonstrate that pro BNP may increase for a variety of causes. Long-term studies with more patients are needed for ST2 to be suitable for clinical use in pediatric patients.

## 1. Introduction

**Figure 1:**
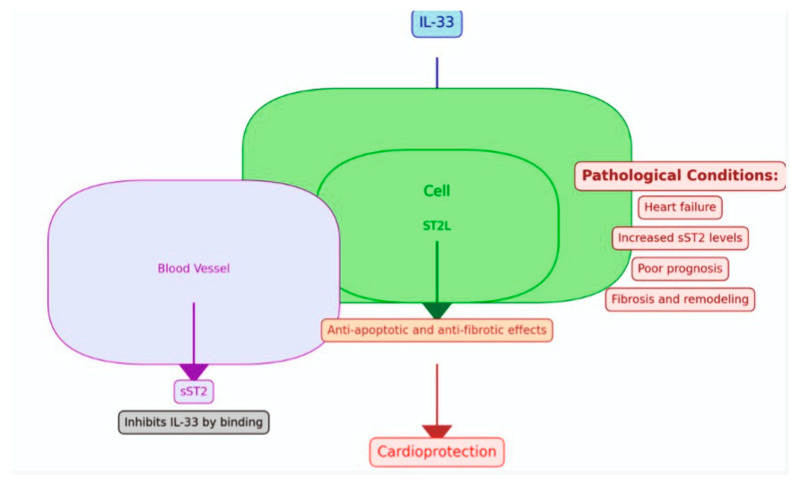
Representation of the role of the suppression of tumorigenicity 2 (ST2) in cardiac function.

Heart failure (HF) in children is a clinical and pathophysiological syndrome that results from ventricular dysfunction, volume, or pressure overload, alone or in combination. It leads to characteristic signs and symptoms such as poor growth, feeding difficulties, respiratory distress, exercise intolerance, and fatigue and is associated with circulatory, neurohormonal, and molecular abnormalities. HF has numerous etiologies that are a consequence of cardiac and non-cardiac disorders, either congenital or acquired [1]. Pediatric heart failure significantly differs from that in adults. The patient population is highly heterogeneous, frequently comprising congenital heart diseases (CHDs) and cardiomyopathies [2]. With the increasing lifespan of patients with congenital heart diseases, the number of patients under follow-up is progressively rising. The management and diagnosis of these patients encompass various challenges. History and symptoms can be confusing due to the influence of accompanying comorbid conditions and assessing functional capacity in young children is difficult. Additionally, echocardiographic and functional evaluations may not be reliable in complex congenital heart diseases and in patients with a systemic right ventricle. For these reasons, the use of biomarkers can provide a more objective assessment. The most commonly used biomarker in children is pro BNP, which is affected by many factors and age. Therefore, the use of new biomarkers is being considered. In the pathophysiology of heart failure, mechanisms such as fibrosis, inflammation, and myocardial stretch are involved. Among these pathways is the suppression of tumorigenicity 2 (ST2), known to be effective in fibrosis, a member of the interleukin-1 (IL-1) receptor family [3,4,5,6,7]. The ligand and soluble versions are its two isoforms. It was first isolated in 1989. It was found to function as an IL-33 ligand in 2005. The IL-33-ST2L ligand complex’s creation offers protection against heart fibrosis and hypertrophy. The formation of this complex is inhibited by soluble ST2, which removes the cardioprotective effect (Figure 1). Unlike NT pro BNP, it is unaffected by age, body mass index, creatinine, hemoglobin, and albumin levels, according to studies performed on individuals with heart failure. The only factor that changes is gender [8]. There was no variation in age or gender among children under the age of fifteen in a research study on healthy children over the age of two. Similar to adults, it has been found to be higher in men than in women above the age of 15 [9]. Similarly, in a study conducted by Caselli et al., it was demonstrated that sST2 levels in healthy children do not change with age [10].

It has been documented that, in adult heart failure patients, soluble ST2 is an independent determinant of mortality and the development of cardiovascular events [11]. The most recent heart failure guideline [12] recommends using it as class 2b in the diagnosing stage. Studies conducted on adults have demonstrated that the prediction rate for cardiovascular events and death following ischemic heart disease is greater than NT pro BNP [13]. The positive predictive value of its use together with biomarkers such as NT pro BNP and hs troponin T was found to be higher [12].

There are few studies evaluating ST2 as a biomarker in children. It was discovered to be an independent predictor of death and morbidity in studies assessing rehospitalization and mortality in young people with congenital heart disease and in the initial postoperative period following congenital heart surgery [14,15,16,17]. In addition, in children with dilated cardiomyopathy, high ST2 values were reported to be positively predictive of anticipated cardiovascular events [18]. In a study involving both adult and pediatric patients with left ventricular assist devices (LVADs), it was observed that ST2 levels decreased after LVAD implantation. However, when comparing the levels before and after the procedure, pediatric patients exhibited higher ST2 levels compared to adults. The reason for this discrepancy has not been elucidated. It has been speculated that the pathophysiology of heart failure may differ between adults and children [19]. Studies examining ST2 in pediatric heart failure patients are lacking, as the literature demonstrates.

The purpose of this study is to determine the prognostic value of ST2 for cardiovascular events in children and adolescents suffering from heart failure. Our secondary goals are to assess ST2 levels in several congenital heart disease groups (cyanotic, acyanotic, obstructive, and congestive heart failure), as well as their differences in predicting major cardiovascular events and correlation with pro BNP.

## 2. Materials and Methods

This study included pediatric patients with cardiac disease, aged 1 month to 18 years. The patients were those with congenital cardiac disease or cardiomyopathy who needed medical care, as well as surgical or interventional treatment in the pediatric cardiology department of Eskisehir Osmangazi University Hospital. Excluded from the trial were individuals who had undergone cardiac surgery within the past month, newborn patients who had chronic renal failure, septic shock, myocardial dysfunction related to cardiopulmonary resuscitation, or for whom consent could not be received. Height, weight, gender, saturation, heart failure classification (Ross or New York Heart Association), drugs taken, electrocardiogram, echocardiography, pro BNP, sST2 values, and the necessity of angiography or surgery during follow-up, hospital stay, lower respiratory tract infection, information on organ dysfunction, arrhythmia, and mortality were all documented for a year. Patients were divided into two groups based on the presence or absence of major adverse cardiac events (MACEs) for comparison. Additionally, the correlation between sST2 levels and pro BNP, as well as their correlation with heart failure scores, was examined.

This study has been approved by Eskişehir Osmangazi University Non-invasive Clinical Research Ethics Committee (code: E-25403353-050.99-174660, date: 2 March 2021) according to the Declaration of Helsinki. Approval was obtained from the patients’ parents to participate in this study. Samples of patients from whom consent was obtained were collected from the patients during routine blood collection for the soluble ST2 level. A 3 mL blood sample was taken, centrifuged, and the serum separated. It was then refrigerated at −80 °C. Pro BNP concentrations were assessed during a routine follow-up in our laboratory. Pro BNP levels were measured using an electrochemiluminescence immunoassay. The human sST2 ELISA measurement kit manufactured by Elabscience Biotechnology (Houston, TX, USA) was used to measure human sST2 from the serum samples. The data were expressed in pg/mL.

The statistical analysis was performed using the IBM SPSS package software version 25. The Shapiro–Wilk test was used to assess how homogeneous the quantitative variables were to the groups’ normal distribution. For variables with regularly distributed data, the t-test was utilized to compare the two groups; for variables with non-normally distributed data, the Mann–Whitney U test was employed. If there were three or more groups to compare, the Kruskal–Wallis test was employed. The factors’ relationships to the MACE were determined using Spearman’s correlation analysis and examples that had analysis results where *p* < 0.05 were considered significant.

## 3. Results

**Figure 2:**
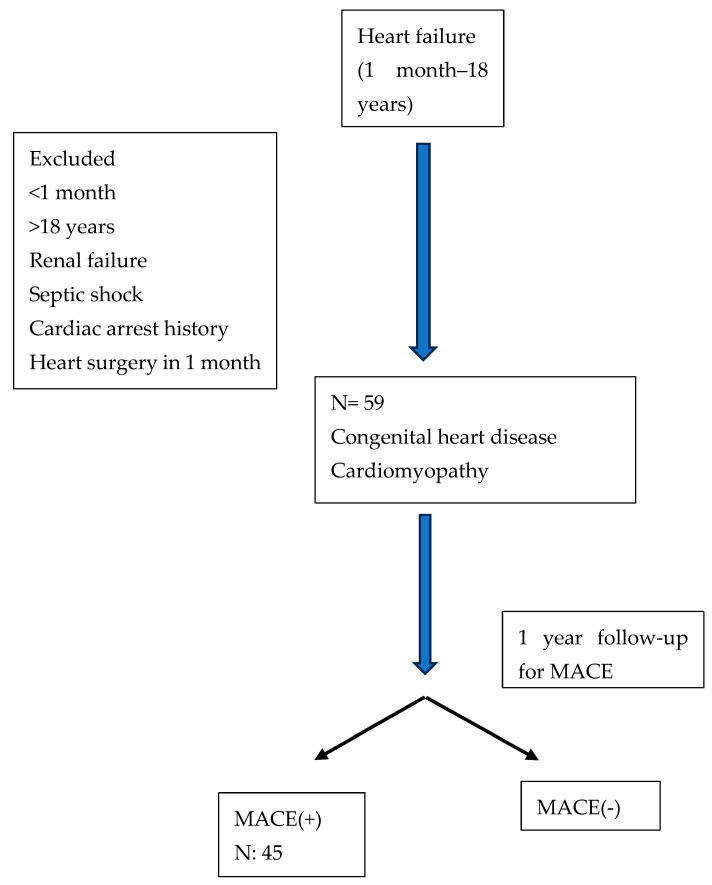
Flowchart of the study.

**Figure 3:**
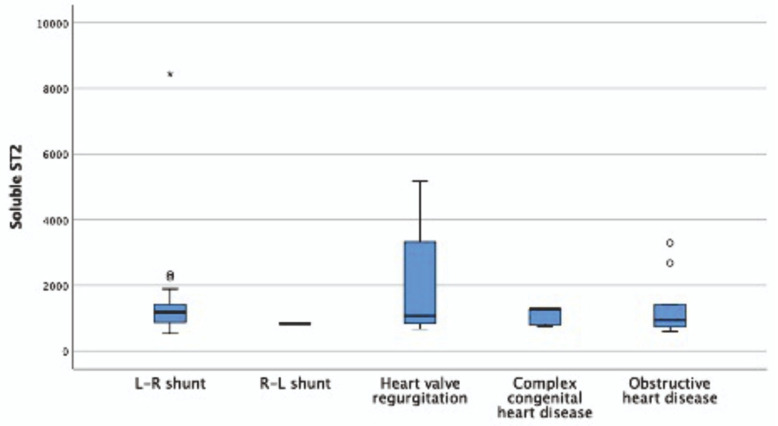
Soluble ST2 levels (pg/mL) according to diagnostic groups.

This study included 59 patients, 27 of whom (46.6%) were male. A total of 55.5 (1–228) months was the median age.Flow chart of study was given Figure 2. The patients’ median height was 114 cm (50–176 cm), and their median body weight was 16 kg (2.6–90 kg). Nine had obstructive type, seven had valve insufficiency, five had complex cyanotic, one patient had heart disease with cyanotic, and 37 patients had hemodynamically important L–R shunts (Figure 3). Of the 59 patients, 45 experienced major cardiovascular events (76.3%) (flow chart). In 24 individuals, a single MACE formed, while in 21 cases, several MACEs developed. Interventional angiography accounts for 50% of these occurrences, growth retardation for 31%, cardiovascular surgery for 25.9%, lower respiratory tract infection for 17.2%, hospitalization requirement for 15.5%, organ dysfunction for 5.2%, arrhythmia for 3.4%, and mortality was listed at 1.7%. Demographic data of the patients are given in Table 1. There was no difference in the age, height, weight, saturation, Qp/Qs, and Ross and NHYA scores of the patients who had MACE compared to those who did not. Pro BNP and sST2 levels did not differ between the groups that experienced MACE and those that did not (Table 2). In contrast, pro BNP was found to be significantly greater (*p* = 0.001, *p* = 0.011, *p* = 0.001, *p* = 0.007, respectively) in patients with lower respiratory tract infection, growth retardation, need for hospitalization, and organ dysfunction when assessed on an event basis (Table 3). Individuals with growth retardation had greater levels of soluble ST2 than individuals without (*p* = 0.037).

The pro BNP level was favorably correlated with the NYHA score (r = 0.648, *p* = 0.023), the soluble ST2 level was positively correlated with the Ross score (r = 0.437, *p* = 0.029), and there was no correlation identified between the pro BNP level and ST2 level (r = 208, *p* = 0.245). The valve disorders group had the highest values for sST2 when the patients were evaluated based on their diagnostic group (Figure 1).

## 4. Discussion

There is a lack of research in the literature investigating the factors influencing sST2 levels for MACE in children with heart failure due to congenital heart disease (CHD) and cardiomyopathy. In our study, although its predictive value for overall MACE development was found to be similar to pro BNP, ST2 levels were significantly higher in children with growth retardation. ST2 levels were notably higher in the valve insufficiency group. A correlation was found with the Ross score, but no association was observed with pro BNP.

In a study assessing 169 patients in the adolescent and adult age group with congenital heart disease, with an average age of 28 years, ST2 levels were found to be significantly higher than the control group. When evaluated in terms of mortality, heart failure, supraventricular tachycardia, atrial fibrillation, and Fontan insufficiency, ST2 was reported to be a strong predictor of mortality. According to reports, in patients with complicated congestive heart failure, its use in conjunction with natriuretic peptides may be predictive of all fatalities [14]. This study was conducted with a larger patient population and included individuals in the older age range compared to our study. In our study, only pediatric patients were included, and no determinants were found for MACE. However, our sample size is small, and the high rate of MACE and the heterogeneous nature of our patient population may be contributing factors. Ragusa et al. monitored ST2 levels in a group of 12 pediatric and 7 adult patients undergoing follow-up before and after LVAD implantation. A decrease in ST2 levels during follow-up was demonstrated. ST2 levels were found to be higher in children than in adults both before and after LVAD implantation [19]. The design of the study differed from ours, and MACE monitoring was not conducted. However, similar to our study, it was found to be highly correlated with clinical heart failure. The reason for the higher levels in children in this study could be related to different unexplained pathophysiological mechanisms. Therefore, further research is needed on the use of ST2 in children. Geenen et al. found, in their analysis of 590 adult patients with congenital heart disease, that it was positively linked with the development of major cardiovascular events, which is similar to the findings of Laqqhan et al.’s study [14,15]. Another study that examined 244 patients who had congenital heart surgery discovered that patients who needed to be readmitted to the hospital within 30 days and experienced death had early postoperative high sST2 levels [16]. Although the design of the study was different from ours, in our study, ST2 did not exhibit a positive predictive value; a similar relationship could not be demonstrated in our study. Martinez et al. evaluated 92 patients with left ventricular dysfunction in their study; ST2 and BNP levels were found to be higher compared to healthy children [20]. However, unlike our study, MACE had not been evaluated and no relationship was found between biomarker levels and functional class.

Additionally, in a study examining specific groups of children with aortic coarctation, preoperative sST2 values were identified as independent predictors of persistent myocardial abnormality. These values were assessed both before and after surgery, with patients followed for a year. Elevated sST2 values were also found to be predictive of cardiovascular events in 94 children with dilated cardiomyopathy [18]. Conversely, no difference in sST2 levels was observed between the control group in a study analyzing patients with pre-clinical and clinical hypertrophic cardiomyopathy; however, changes were noted in pro BNP and troponin levels [19]. In another study involving twelve patients with Duchenne muscular dystrophy (DMD), the levels of ST2, logMMP9, Gal 3, TLR4, Lep, TNC, BNP, and CRP were evaluated. It was found that both groups with and without cardiomyopathy (CMP) had higher levels of soluble ST2 compared to the healthy control group. Furthermore, when comparing DMD patients with CMP to those without CMP, a statistically significant difference in ST2 levels was observed, whereas other biomarkers showed no differences [21]. Despite the fact that the group in our study was not homogeneous, and we did not compare with a healthy control group, the group with valve disease in particular had a higher sST2 level. The correlation between the pro BNP level and ST2 could not be demonstrated in our study, similar to many studies [14,15]. Conversely, pro BNP was discovered to be significantly higher in the MACE group, particularly in patients who needed interventional angiography, had a lower respiratory tract infection, were hospitalized, or had growth retardation. Only individuals with growth retardation in the MACE group had higher levels of soluble ST2 than those without. A correlation between the ST2 level and Ross score was found. This correlation is supported by the finding that patients with growth retardation have higher Ross scores and higher ST2 levels. Pro BNP is believed to be influenced by numerous causes, in contrast to sST2 [14].

The limited number of patients, the brief follow-up time, the heterogeneity of the patient group, and the relatively high MACE rate resulting from the exclusion of congenital cardiac disorders without heart failure are the limitations of our study, as well as the absence of healthy control comparisons and single measurement limits. Another reason for the lack of predictive value of ST2 in our study could be the high rate of MACE.

As a result, in our study, sST2 levels were notably elevated, particularly in the group with growth retardation and valve insufficiency. It was found to be correlated with the Ross score but not with pro BNP levels. Despite its association with clinical heart failure, sST2 exhibited poor prognostic value for major adverse cardiovascular events (MACEs). On the other hand, pro BNP levels may increase in response to various factors, as evidenced by elevations observed in patients with lower respiratory tract infections, growth retardation, hospitalizations, and organ dysfunction. Longer-term trials with a larger patient population are required to evaluate whether sST2 is appropriate for clinical use in pediatric patients.

## Figures and Tables

**Table 1:** Demographic data of the patients.

		Median (Min–Max)
Age (month)		55.5 (1–228)
Height (cm)		114 (50–176)
Weight (kg)		16.00 (2.6–90)
Gender	F	53.4%
	M	46.6%
Oxygen saturation (%)		98 (74–100)
PAP (mmHg)		20 (10–99)
Qp/Qs		1.85 (1–2.6)
Pro BNP (pg/mL)		313 (17–22,243)
sST2 (pg/mL)		1110 (530–8440)
MACE		76.3%
Lower respiratory tract infection		17.2%
Arrhythmia		3.4%
Interventional Angiography		50%
Cardiovascular surgery		25.9%
Growth retardation		31%
Hospitalization		15.5%
Organ dysfunction		5.2%
Mortality		1.7%

PAP: pulmonary artery pressure.

**Table 2:** Comparison of data according to the presence of MACE.

	MACE (−)	MACE (+)	*p*
Age (month)	69.0 (1.5–204)	53.0 (1–228)	0.592
Height (cm)	139 (52–160)	113 (50–176)	0.312
Weight (kg)	15.25 (2.9–87)	16.40 (2.6–90)	0.702
Oxygen saturation (%)	98 (94–100)	97 (74–100)	0.214
PAP (mmHg)	47 (23–70)	20 (10–99)	0.142
Qp/Qs	1.00	1.90 (1.1–2.6)	0.095
Ross score	3 (1–6)	4 (0–10)	0.233
NYHA stage	2 (2)	2 (2–3)	0.303
Pro BNP	258 (20–9204)	314 (17–22,243)	0.808
sST2 (pg/mL)	955 (665–3290)	1245 (530–8440)	0.277

PAP: pulmonary artery pressure; MACE: major cardiovascular event.

**Table 3:** Comparison of pro BNP and sST2 levels according to MACE subgroups.

		Pro BNP (pg/mL) Median (Min–Max)	*p*	ST2 (pg/mL) Median (Min–Max)	*p*
**MACE**	−	2588 (20–9204)	0.808	955 (665–3290)	0.277
	+	314 (17–22,243)		1245 (530–8440)	
Lower respiratory tract infection	−	198 (17–9204)	**0.001**	1040 (530–3290)	0.064
	+	5841 (313–22,243)		1313 (800–8440)	
Arrhythmia	−	286 (17–22,243)	0.483	1110 (530–8440)	0.701
	+	3432 (217–6646)		2965 (800–5130)	
Interventional angiography	−	742 (20–22,243)	**0.019**	905 (560–8440)	0.286
	+	193 (17–1354)		1245 (530–2675)	
Cardiovascular surgery	−	245 (17–9204)	0.096	1040 (530–5165)	0.702
	+	2853 (148–22,243)		1280 (560−8440)	
Growth retardation	−	196 (20–9204)	**0.011**	1023 (530−2675)	**0.037**
	+	807 (17–22,243)		1313 (700–8440)	
Hospitalization	−	198 (17–9204)	**0.001**	1040 (530–3290)	0.107
	+	5841 (313–22,243)		1280 (800–8440)	
Organ dysfunction	−	258 (17–9204)	**0.007**	1110 (530−8440)	0.765
	+	14,023 (6646–22,243)		800 (800–2335)	
Mortality	−	313 (17–14,023)	0.093	1075 (530–8440)	0.143
	+	22,243 (22,243–22,243)		2335	

*p* < 0.05 was considered significant (bold).

## Data Availability

The original contributions presented in the study are included in the article, further inquiries can be directed to the corresponding author.
